# Decrease in Prosaposin in the Dystrophic mdx Mouse Brain

**DOI:** 10.1371/journal.pone.0080032

**Published:** 2013-11-14

**Authors:** Hui-ling Gao, Cheng Li, Hiroaki Nabeka, Tetsuya Shimokawa, Naoto Kobayashi, Shouichiro Saito, Zhan-You Wang, Ya-ming Cao, Seiji Matsuda

**Affiliations:** 1 Department of Anatomy and Embryology, Ehime University Graduate School of Medicine, Toon, Ehime, Japan; 2 Medical Education Center, Ehime University Graduate School of Medicine, Toon, Ehime, Japan; 3 Laboratory of Veterinary Anatomy, Faculty of Applied Biological Sciences, Gifu University, Yanagido, Gifu, Japan; 4 College of Life and Health Sciences, Northeastern University, Shenyang, China; 5 Department of Immunology, China Medical University, Shenyang, China; Hertie Institute for Clinical Brain Research and German Center for Neurodegenerative Diseases, Germany

## Abstract

**Background:**

Duchenne muscular dystrophy caused by a mutation in the X-linked dystrophin gene induces metabolic and structural disorders in the brain. A lack of dystrophin in brain structures is involved in impaired cognitive function. Prosaposin (PS), a neurotrophic factor, is abundant in the choroid plexus and various brain regions. We investigated whether PS serves as a link between dystrophin loss and gross and/or ultrastructural brain abnormalities.

**Methodology/Principal Findings:**

The distribution of PS in the brains of juvenile and adult mdx mice was investigated by immunochemistry, Western blotting, and *in* *situ* hybridization. Immunochemistry revealed lower levels of PS in the cytoplasm of neurons of the cerebral cortex, hippocampus, cerebellum, and choroid plexus in mdx mice. Western blotting confirmed that PS levels were lower in these brain regions in both juveniles and adults. Even with low PS production in the choroids plexus, there was no significant PS decrease in cerebrospinal fluid (CSF). *In* *situ* hybridization revealed that the primary form of PS mRNA in both normal and mdx mice was Pro+9, a secretory-type PS, and the hybridization signals for Pro+9 in the above-mentioned brain regions were weaker in mdx mice than in normal mice. We also investigated mitogen-activated protein kinase signalling. Stronger activation of ERK1/2 was observed in mdx mice, ERK1/2 activity was positively correlated with PS activity, and exogenous PS18 stimulated both p-ERK1/2 and PS in SH-SY5Y cells.

**Conclusions/Significance:**

Low levels of PS and its receptors suggest the participation of PS in some pathological changes in the brains of mdx mice.

## Introduction

Prosaposin (PS) is a multifunctional protein involved in a variety of biological processes, where it is either transported to lysosomes or secreted into the extracellular space [1-3]. In lysosomes, PS is proteolytically processed to generate four sphingolipid activator proteins, known as saposins A to D, which are required for hydrolysis of sphingolipids by several lysosomal exohydrolases. Many functions have been attributed to secreted PS, which is reportedly a trophic factor in the nervous and reproductive systems, being present in milk and cerebrospinal and seminal fluids [4-8]. 

The PS gene contains 15 exons. It is transcribed into several mRNAs, resulting from alternative splicing of the 9-bp exon 8 [9]. *In situ* hybridization has shown abundant PS expression in the epithelial cells of the choroid plexus and various grey matter areas, including the cortex and hippocampus [10,11]. Besides its role as the precursor protein of saposins, PS is also a neurotrophic factor [12] capable of inducing neural differentiation and preventing cell death. A neurotrophic sequence has been identified in 14 amino acids located in the N-terminal part of saposin C [13] and has been attributed to PS neurotrophic activity [14,15]. Moreover, a PS-derived 18-mer peptide attenuates dopaminergic neurotoxicity by downregulating c-Jun, BAX, and caspase-3, and upregulating Bcl-2 [4].

Duchene muscular dystrophy (DMD) is a fatal genetic disease caused by mutations in the DMD gene, leading to dystrophin deficiency [16,17]. DMD is caused by a mutation in the X-linked dystrophin gene [18]; it is a recessive genetic disease characterised by alterations in the neuromuscular system, and metabolic and structural disorders of the central nervous system (CNS), which cause mental retardation and metabolic damage [19]. While muscle wasting is prominent, the CNS is also affected in DMD, with non-progressive intellectual and/or cognitive impairment being observed in about one-third of patients with DMD [20-22]. 

The dystrophin-deficient mdx mouse is a model of human DMD [23]. In the brain, the cerebral cortex, cerebellum and areas CA1-CA3 of the hippocampus are regions in which dystrophin is known to be expressed [24-26]. Brain dystrophin is enriched in the postsynaptic densities of pyramidal neurons, specialised regions of the subsynaptic cytoskeletal network that are critical for synaptic transmission and plasticity. Loss of dystrophin, together with a consequent abnormality of the dystrophin-associated protein complex (DAPC), gives rise to a complex syndrome of progressive skeletal and cardiac myopathy and mental retardation. Recently, we reported low levels of PS in muscles in mdx mice compared with C57BL/10 mice [7].

Whether PS is a link between dystrophin loss and gross and/or ultrastructural brain abnormalities remains unclear. In this study, we examined the expression of PS at the protein and transcriptional levels in the CNS of mdx mouse by immunochemistry, Western blotting and *in situ* hybridization. 

## Results

### PS protein expression in mdx and C57BL/10 mice

**Figure 1:**
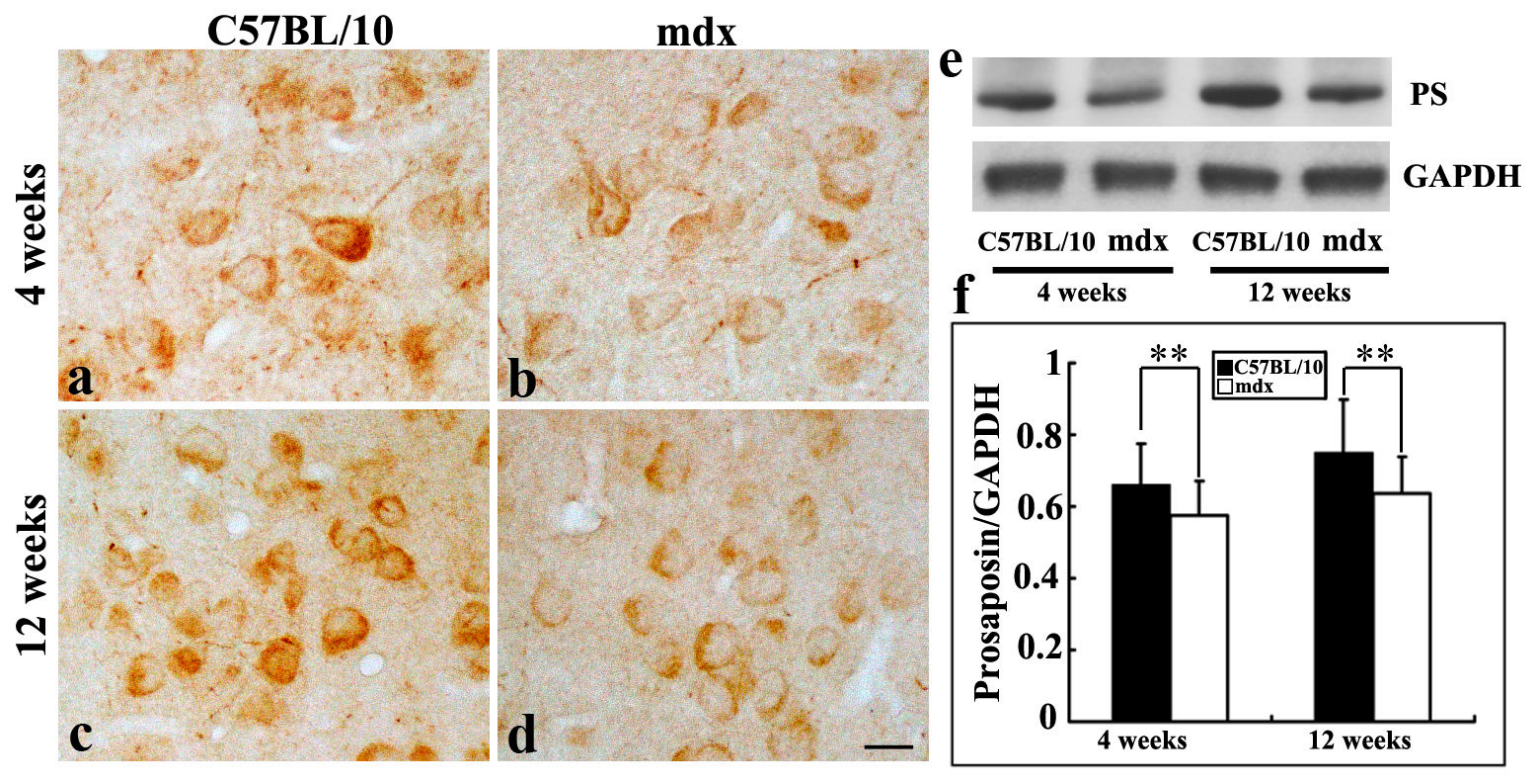
PS in the cerebral cortex of C57BL/10 and mdx mice, as detected by immunochemistry and Western blotting. **a**–**d**: Immunoreactivity is present in the somas and primary dendrites of most neurons in the cerebral cortex in juvenile (**a**, **b**) and adult (**c**, **d**) C57BL/10 and mdx mice. Bars = 20 μm. **e**: Western blot analysis showing PS as a 65-kDa protein in the hippocampus of juvenile and adult C57BL/10 and mdx mice. **f**: Relative PS protein levels in mdx and control mice at 4 and 12 weeks, as determined by densitometry. Densitometric values were normalized using GAPDH as an internal control. Results were analyzed using Fisher’s post hoc test (**p < 0.01).

**Figure 2:**
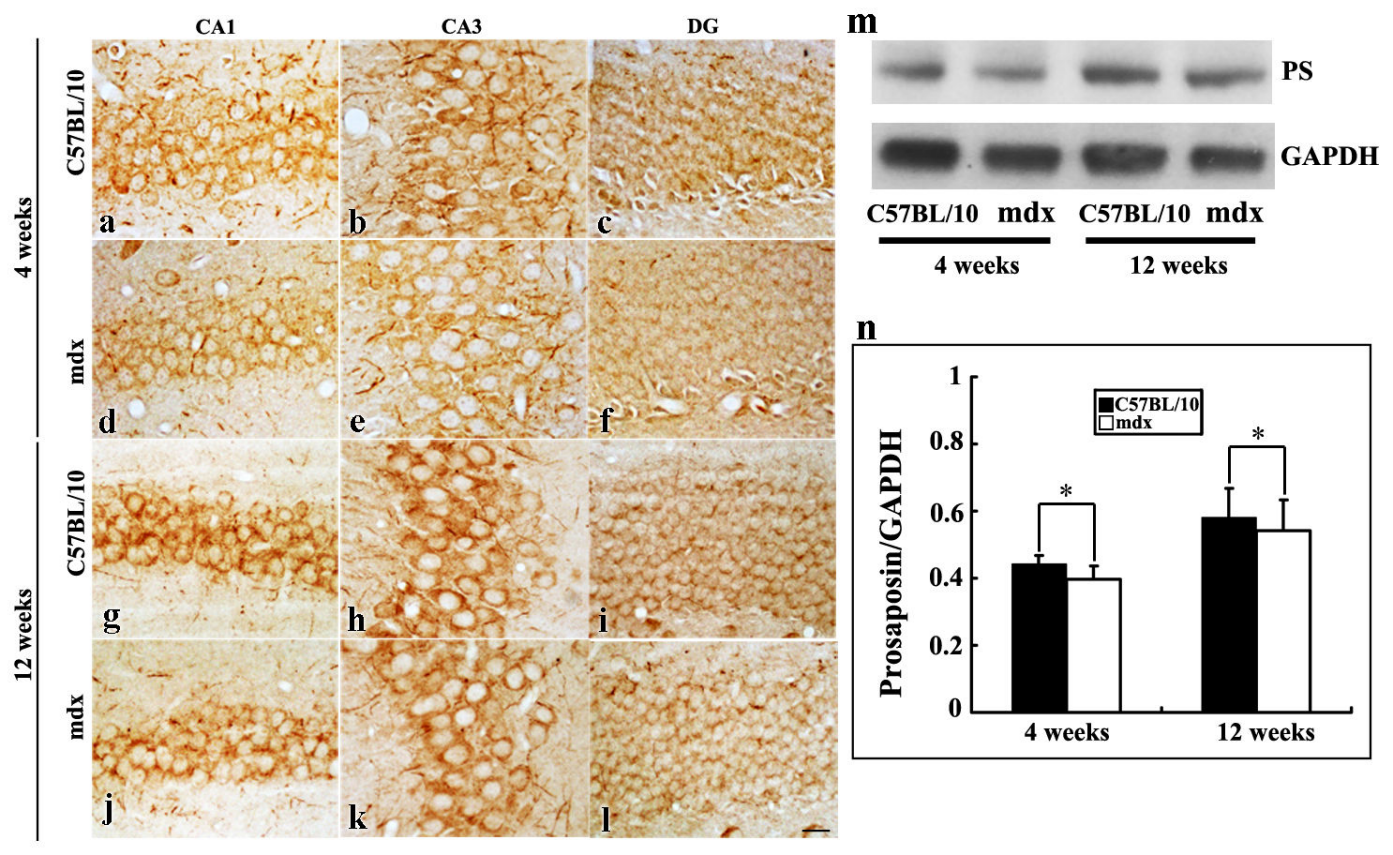
PS in the hippocampus of C57BL/10 and mdx mice, as detected by immunochemistry and Western blotting. **a**–**l**: In the CA1, CA3 and DG areas of the hippocampus, PS immunoreactivity was found in the somas and primary dendrites in both juvenile (**a**–**f**) and adult (**g**–**l**) C57BL/10 and mdx mice. Bars = 20 μm. **m**: Western blot analysis showing PS as a 65-kDa protein in the hippocampus of juvenile and adult C57BL/10 and mdx mice. **n**: Relative protein levels, as determined by densitometry. Densitometric values were normalized using GAPDH as an internal control. Results were analyzed using Fisher’s post hoc test (**p < 0.01).

**Figure 3:**
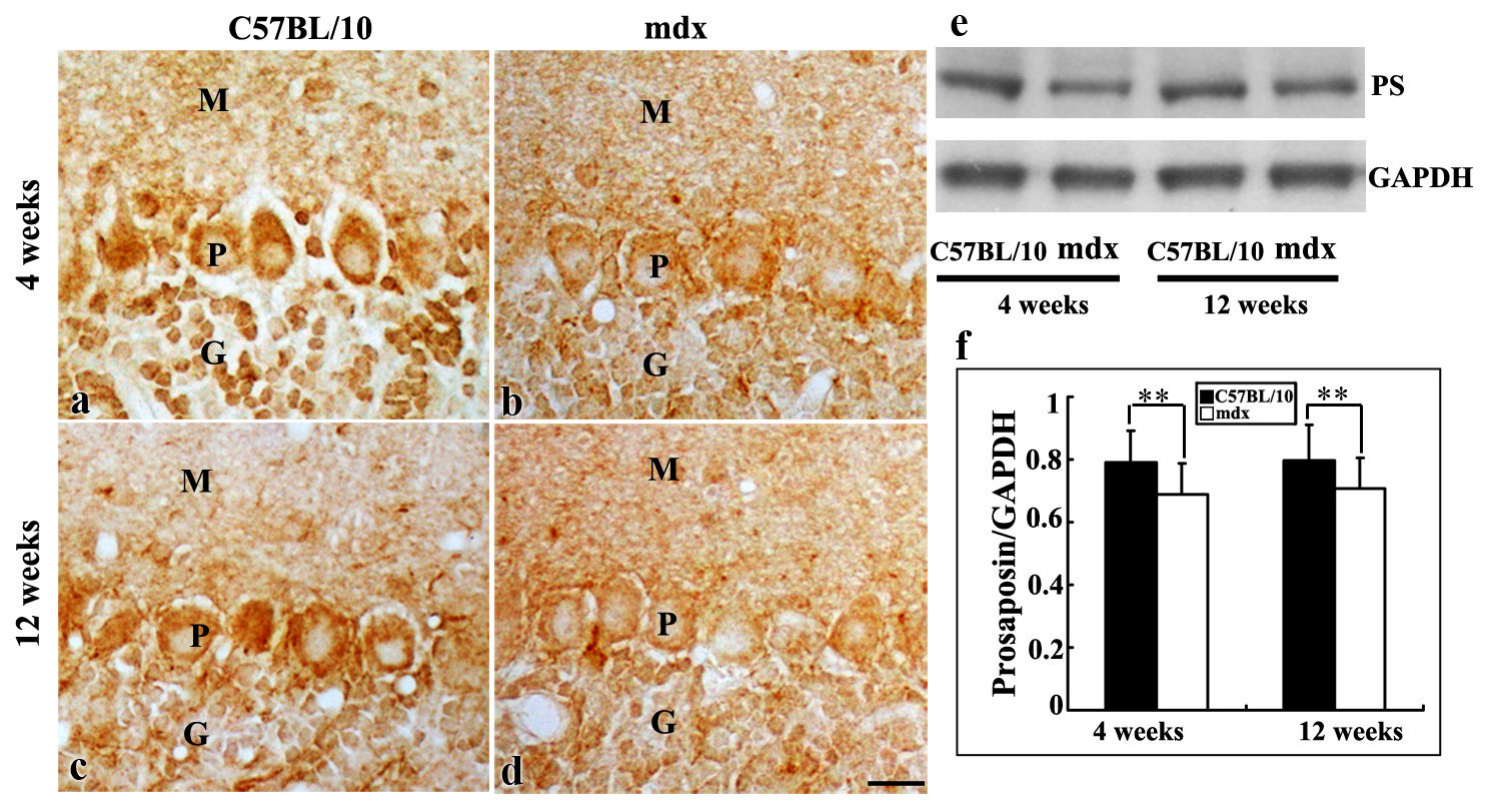
PS in the cerebellum of C57BL/10 and mdx mice, as detected by immunochemistry and Western blotting. **a**–**d**: Light micrographs showing PS immunoreactivity in Purkinje and granule cell bodies in juvenile (**a**, **b**) and adult (**c**, **d**) C57BL/10 and mdx mice. M, molecular layer; P, Purkinje cell layer; G, granule cell layer. Bars = 20 μm. **e**: Western blot analysis showing PS as a 65-kDa protein in the cerebellum of juvenile and adult C57BL/10 and mdx mice. Densitometric analysis showed that PS levels were substantially lower in mdx mice than in control mice both at 4 and 12 weeks (**f**). The results were analyzed by Fisher’s post hoc test (*p < 0.01).

To investigate PS protein expression in the mouse cerebral cortex, hippocampus and cerebellum, immunohistochemical and Western blot analyses were performed. PS-like immunoreactivity was observed in different brain regions in juvenile and adult mice. The PS staining exhibited a granular pattern in the cytoplasm of neurons (Figures 1a–d, 2a–l, 3a–d). Western blot analysis was performed to investigate PS protein expression in mdx and C57BL/10 mice aged 4 and 12 weeks. Since the anti-PS antibody was obtained from the intermediate sequence between saposin C and D, it only reacts with PS and not with saposins in immunochemistry and Western blotting. As expected, PS protein was detected as a band of 65 kDa (Figures 1e, 2m, 3e).

In the cerebral cortex of C57BL/10 mice, PS immunoreactivity was predominantly present in the somas and primary dendrites of most neurons in animals aged 4 and 12 weeks (Figure 1a–d). In the mdx mice, the PS staining pattern was similar, but the staining in the cortex was weaker in mdx mice aged 4 and 12 weeks (Figure 1b, d). This was confirmed by Western blotting (Figure 1e). Densitometry of PS-immunoreactive bands showed that PS levels in the cerebral cortex were significantly lower in mdx mice than in control mice at 4 weeks (0.56 ± 0.09 vs. 0.66 ± 0.11, *p* < 0.01; Figure 1f) and 12 weeks (0.63 ± 0.10 vs. 0.75 ± 0.11, *p* < 0.01; Figure 1f).

In the CA1, CA3 and DG areas of the hippocampus, PS grains were primarily observed in the cytoplasm (Figure 2a–l) and in some big neurites extending from PS-positive neuronal somata in the CA1 and CA3 areas (Figure 2a, b, d, e, g, h, j, k) in C57BL/10 and mdx mice aged 4 and 12 weeks. PS immunoreactivity was weaker in mdx mice than in C57BL/10 mice. In the hippocampus, PS expression was lower in mdx mice than in C57BL/10 mice at both 4 weeks (0.39 ± 0.03 vs. 0.50 ± 0.02, *p* < 0.05) and 12 weeks (0.50 ± 0.09 vs. 0.58 ± 0.08, *p* < 0.05; Figure 2m, n).

In the cerebellum, PS immunoreactivity was predominantly observed in the somas of Purkinje neurons. A few PS-positive neuronal somas were seen in the molecular layer. In the granule cell layer, PS immunoreactivity was observed in the cytoplasm of granule cells (Figure 3a–d). In mdx mice, PS immunoreactivity was much weaker in the Purkinje cell layer and granule cell layer, but not in the molecular layer, compared with control mice. Similar results were obtained by Western blotting. PS levels were significantly lower in mdx mice than in control mice at 4 weeks (0.65 ± 0.09 vs. 0.79 ± 0.10, *p* < 0.01) and 12 weeks (0.70 ± 0.09 vs. 0.85 ± 0.11, *p* < 0.01; Figure 3e, f).

### PS mRNA expression in mdx and C57BL/10 mice

**Figure 4:**
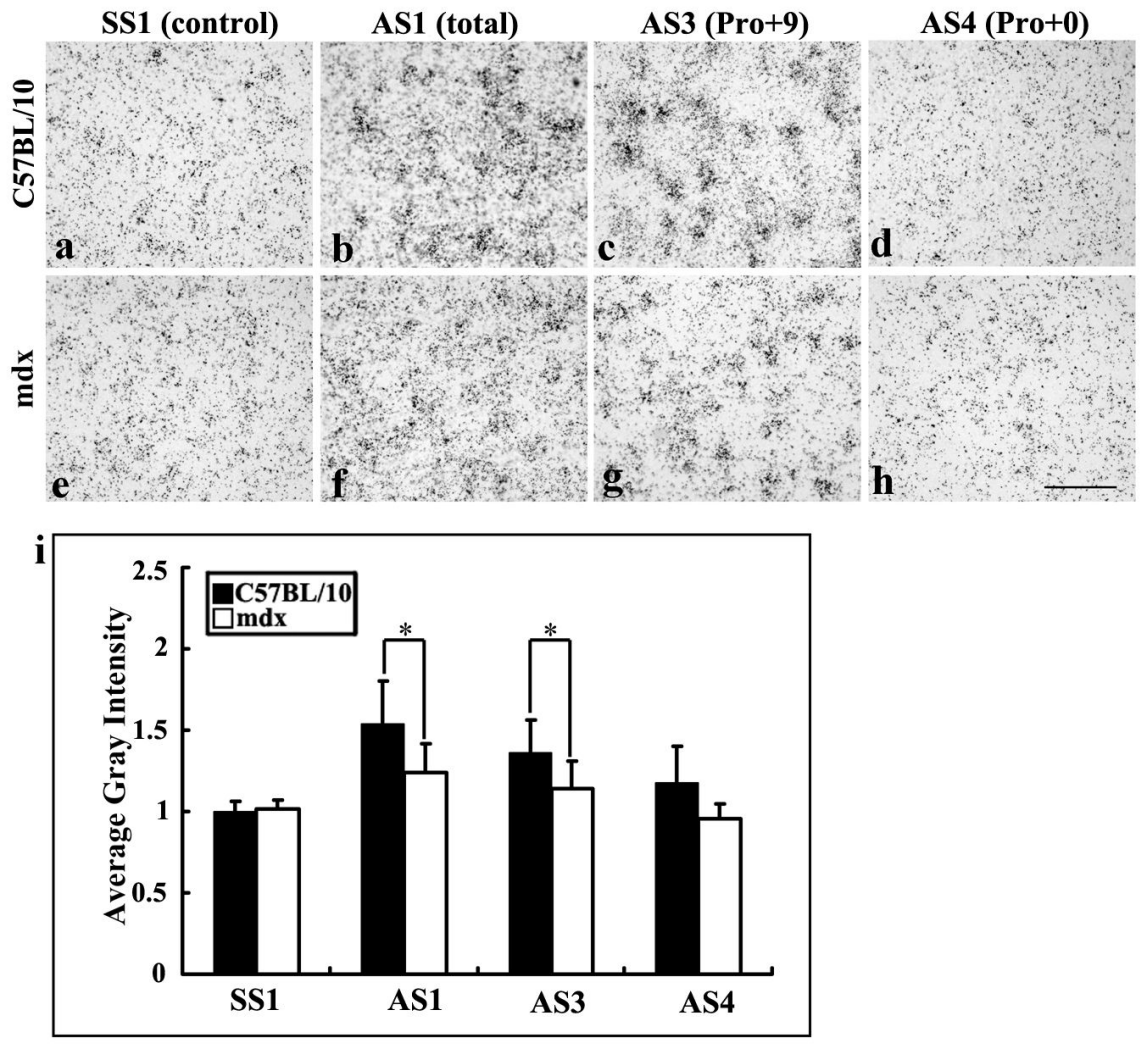
PS mRNA expression in the cerebral cortex of C57BL/10 and mdx mice at 4 weeks, as shown by *in* *situ* hybridization with [^35^S]-labelled antisense oligonucleotide probes. **b**, **f**: Detection of total mRNA with AS1. **c**, **g**: Detection of exon 8-containing PS mRNA with AS3. **d**, **h**: Detection of exon 8-excluded PS mRNA with AS4. **a**, **e**: the sense probe SS1 (used as a control). Positive reactions (labelled with concentrated silver grains) can be identified in the neurons of control and mdx mice. The hybridization signals for total mRNA (AS1) and Pro+9 mRNA (AS3) were weaker in mdx mice than in C57BL/10 mice. No obvious reactivity was observed for AS4 and SS1. Results were analyzed by ANOVA followed by Fisher’s post hoc test (*p < 0.05). Bars = 10 μm.

**Figure 5:**
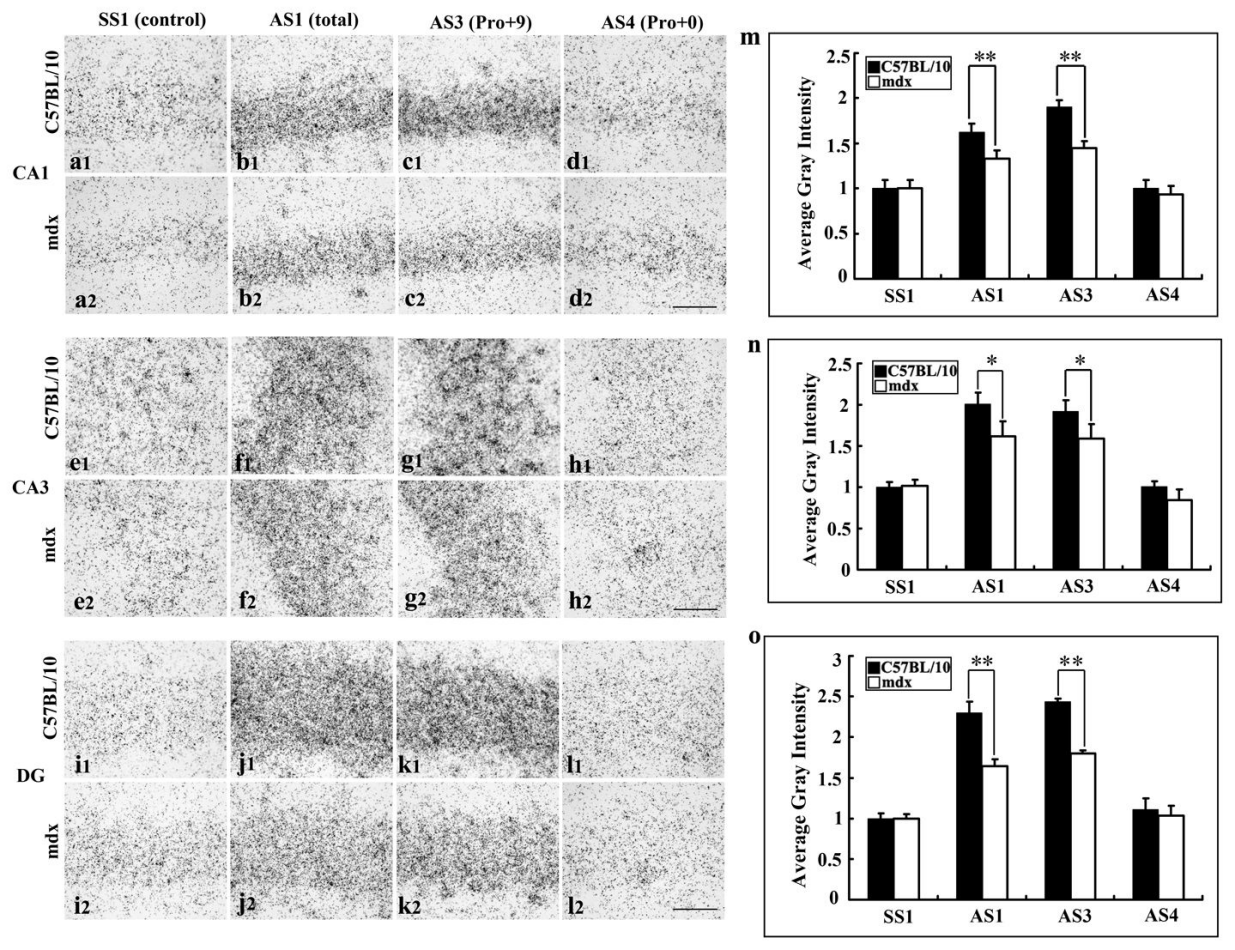
PS mRNA expression in the hippocampal regions of C57BL/10 and mdx mice at 4 weeks. **a1-d2**: CA1. e**1-h2**: CA3. i**1-**l **2**: Dentate gyrus (DG). Total PS mRNA expression was detected by *in* *situ* hybridization using AS1, and the signals in the hippocampal regions CA1, CA3 and DG were weaker in mdx mice than in C57BL/10 mice (**b1, b2, f1, f2, j1, j2**). Pro+9 mRNA expression in the hippocampal regions CA1 (**c1, c2**), CA3 (**g1, g2**) and DG (**k1, k2**) decreased in mdx mice. The intensity of the hybridization signal for Pro+0 was weak. The control group labelled with the sense probe SS1 showed no specific signals. **m**, **n**, **o**: Results were analyzed by ANOVA followed by Fisher’s post hoc test (*p < 0.05, **p < 0.01). Bars = 10 μm.

**Figure 6:**
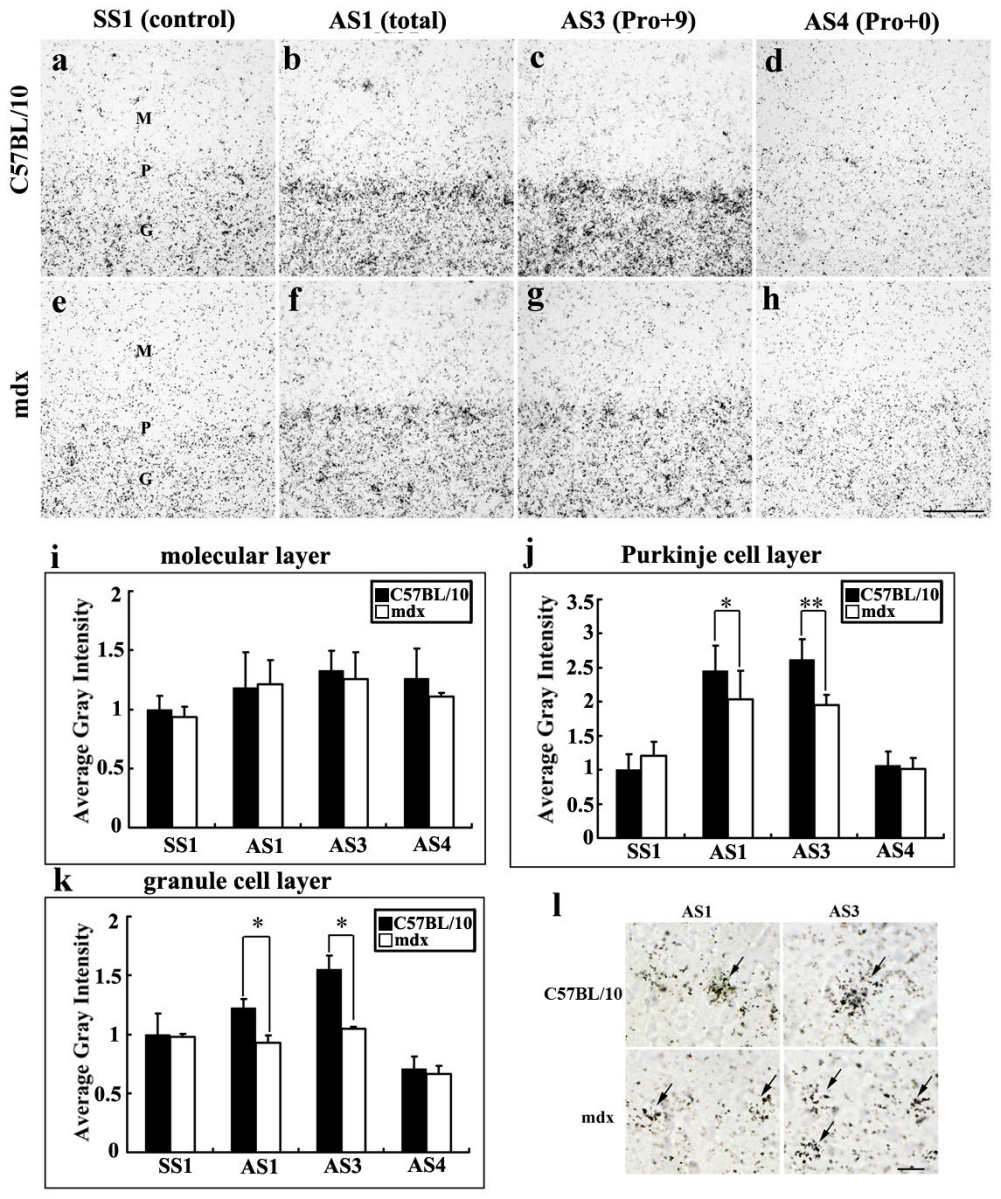
PS mRNA expression in the cerebellum in C57BL/10 (a–d) and mdx mice (e–h) at 4 weeks. **b**, **f**: Detection of total mRNA with AS1. **c**, **g**: Detection of exon 8-containing PS mRNA with AS3. **d**, **h**: Detection of exon 8-excluded PS mRNA with AS4. **a**, **e**: the sense probe SS1 (used as a control). In the Purkinje cell layer (**j**) and granular cell layer (**k**) of the cerebellum, the total PS mRNA (AS1) and Pro+9 mRNA (AS3) levels were lower in mdx mice than in C57BL/10 mice. (**i**) In the molecular cell layer, no differences were observed in total PS mRNA and Pro+9 mRNA levels between mdx and C57BL/10 mice. (**l**) At higher magnification, the expression of PS in interneurons was detected. AS1 and AS3 showed intense signals in C57BL/10 mice, but weak ones in mdx mice. No obvious signals were detected for AS4 and SS1. Results were analyzed by ANOVA followed by Fisher’s post hoc test and are presented as a histogram (*p < 0.05). Bars = 50 μm.

To determine the spatial expression pattern of PS isoforms at the single-cell level, we analyzed brain sections from juvenile mice (age 4 weeks) by *in situ* hybridization with oligonucleotide probes encoding PS sequences. The analysis revealed a similar distribution pattern (Figures 4–6). Numerous labelled neurons were observed in various brain regions, including the cerebral cortex, hippocampus and cerebellum, in both mdx and normal mice. To determine whether PS mRNA expression was different between mdx and C57BL/10 mice, we measured the intensities of hybridization signals in brain sections using ImageJ software, and analyzed the data by Fisher’s post hoc test (Figures 4i, 5m–o, 6i–k). In the sections labelled with one of the four probes, the signals showed different intensities. The control group, labelled with the sense probe SS1 showed weak signals (Figure 4a, e). The intensities of the hybridization signals for Pro+0 (AS4) were also weaker (Figure 4d, h). In the cerebral cortex, the hybridization signals for total mRNA (AS1, Pro+9 and Pro+0) for mdx mice (1.23 ± 0.17) were weaker than those for C57BL/10 mice (1.54 ± 0.26; Figure 4b, f, i). The hybridization signal for Pro+9 mRNA (AS3, encoding secretory-type PS) was also weaker in mdx mice than in C57BL/10 mice (1.13 ± 0.17 vs. 1.56 ± 0.20; Figure 4c, g, i). Statistically significant decreases in total and Pro+9 signals were detected in mdx mice (Figure 4i), indicating that the decrease in PS levels in mdx mice is mainly due to a decrease in Pro+9 mRNA expression.

Also in the hippocampal CA1, CA3 and DG areas in mdx and C57BL/10 mice, AS1 (Pro+9 and Pro+0) and AS3 (Pro+9) showed strong signals compared with SS1 (control) and AS4 (Pro+0; Figure 5). In CA1, the intensity of AS1 was significantly lower in mdx mice than in C57BL/10 mice (1.32 ± 0.09 vs. 1.62 ± 0.12, *p* < 0.01; Figure 5b1, b2, m). Moreover, the distribution pattern of AS3 in mdx mice was similar to that of AS1, and its intensity was lower in mdx mice compared with C57BL/10 mice (1.45 ± 0.07 vs. 1.90 ± 0.10, *p* < 0.01; Figure 5c1, c2, m). In CA3, AS1 was lower in mdx mice compared with C57BL/10 mice (1.51 ± 0.17 vs. 2.01 ± 0.13, *p* < 0.05; Figure 5f1, f2, n) and AS3 was also lower in mdx mice than in C57BL/10 mice (1.56 ± 0.16 vs. 1.98 ± 0.13, *p* < 0.05; Figure 5g1, g2, n). In DG areas, AS1 and AS3 were also lower in mdx mice than in C57BL/10 mice (AS1: 1.79 ± 0.07 vs. 2.29 ± 0.13, *p* < 0.01; Figure 5j1, j2; AS3: 1.75 ± 0.04 vs. 2.43 ± 0.03, *p* < 0 .01; Figure 5k1, k2, o). AS4 (Pro+0) showed very weak reactions (Figure 5d1, d2, h1, h2, l 1, l2), similar to those of the control group labelled with the sense probe SS1 (Figure 5a1, a2, e1, e2, i1, i2). 

In the Purkinje cell layer of the cerebellum, the total PS mRNA (AS1: Pro+9 and Pro+0) and AS3 (Pro+9) signals were lower in mdx mice than in C57BL/10 mice (AS1: 2.03 ± 0.22 vs. 2.45 ± 0.26; AS3: 1.94 ± 0.15 vs. 2.62 ± 0.22; Figure 6b, c, f, g, j). In the granular cell layer, the intensity of AS1 was lower in mdx mice than in C57BL/10 mice (0.92 ± 0.06 vs. 1.22 ± 0.07, *p* < 0.05; Figure 6b, c, j), and that of AS3 was considerably lower in mdx mice than in C57BL/10 mice (1.04 ± 0.02 vs. 1.55 ± 0.11, *p* < 0.01; Figure 6f, g, k). In the molecular layer, both AS1 and AS3 showed no big differences in average intensity between mdx and C57BL/10 mice (*p* > 0.05; Figure 6b, c, f, g, i) when scanned at 400× magnification. However, sections observed under a higher magnification showed lower expression of AS1 and AS3 in the interneurons in the molecular layer in mdx mice than in C57BL/10 mice (Figure 6l). AS4 showed very weak reactions (Figure 6d, h), similar to those in the control group labelled with SS1 (Figure 6a, e). 

### MAP kinase activity in mdx and C57BL/10 mice

Activation of the MAPK pathway by PS, saposin C or TX14A has been reported in neuronal- or glial-derived cells such as PC12, Schwann and neuroblastoma cells [27,28]. To determine whether MAPK signalling is related to PS in mdx neurons, we analyzed three components (p38 MAP kinase, ERK1/2 and JNK1/2) of the MAP kinase cascades by Western blotting.

**Figure 7:**
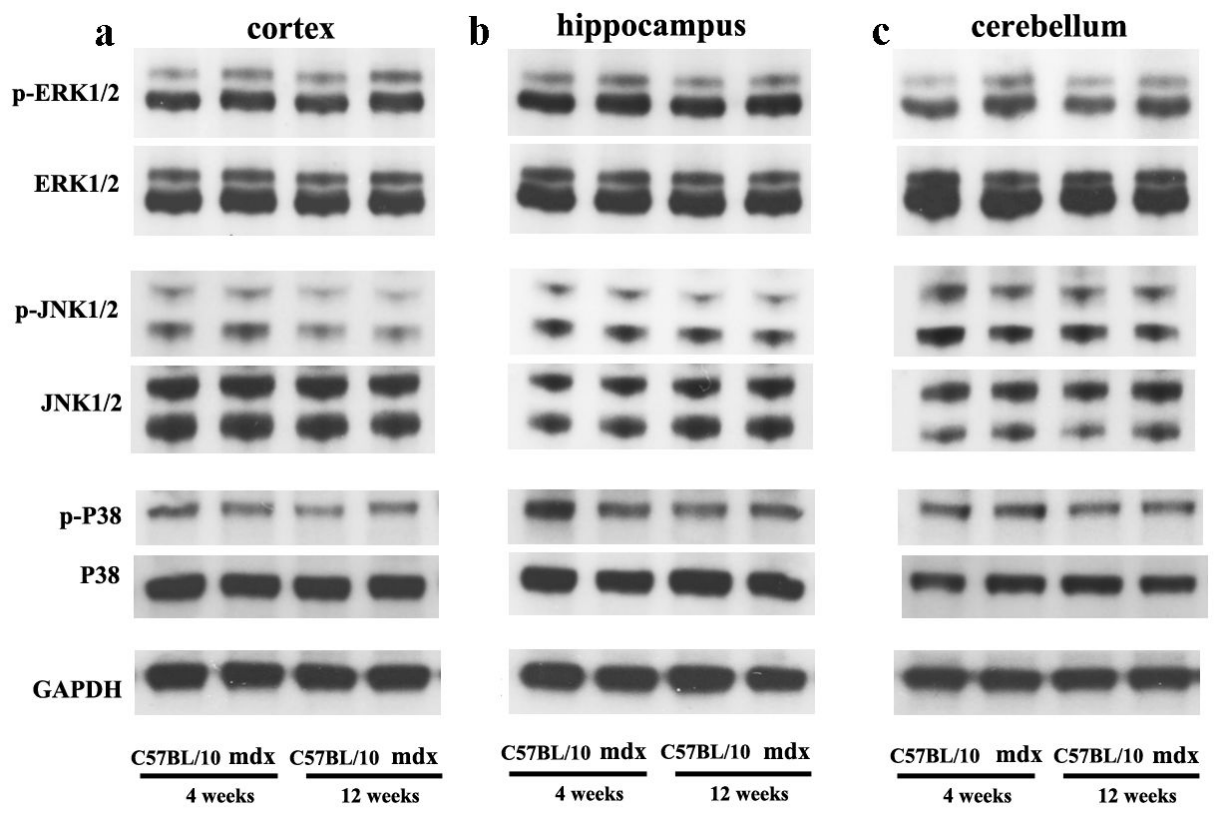
MAP kinases in mdx and C57BL/10 mice aged 4 and 12 weeks. **a**: Cerebral cortex. **b**: Hippocampus. **c**: Cerebellum. Three components (p38 MAP kinase, ERK1/2 and JNK1/2) of MAP kinase cascades were analyzed by Western blotting. GAPDH was used as a control for protein loading.

ERK1 and ERK2 were dually phosphorylated at T202/Y204 and T185/Y187, respectively, and were detected as double bands at 44 kDa (p-ERK1) and 42 kDa (p-ERK2). Western blotting showed that the level of p-ERK1/2 in the brain was higher in mdx mice (ages 4 and 12 weeks) than in C57BL/10 mice of the same age (Figure 7a–c). No differences in the phosphorylation levels of p-JNK1/2 or p38 MAPK (p-p38) were observed between juvenile/adult mdx mice and C57BL/10 mice of the same age (Figure 7a–c). No changes in the core levels (non-phosphorylated forms) of these proteins were detected. 

**Figure 8:**
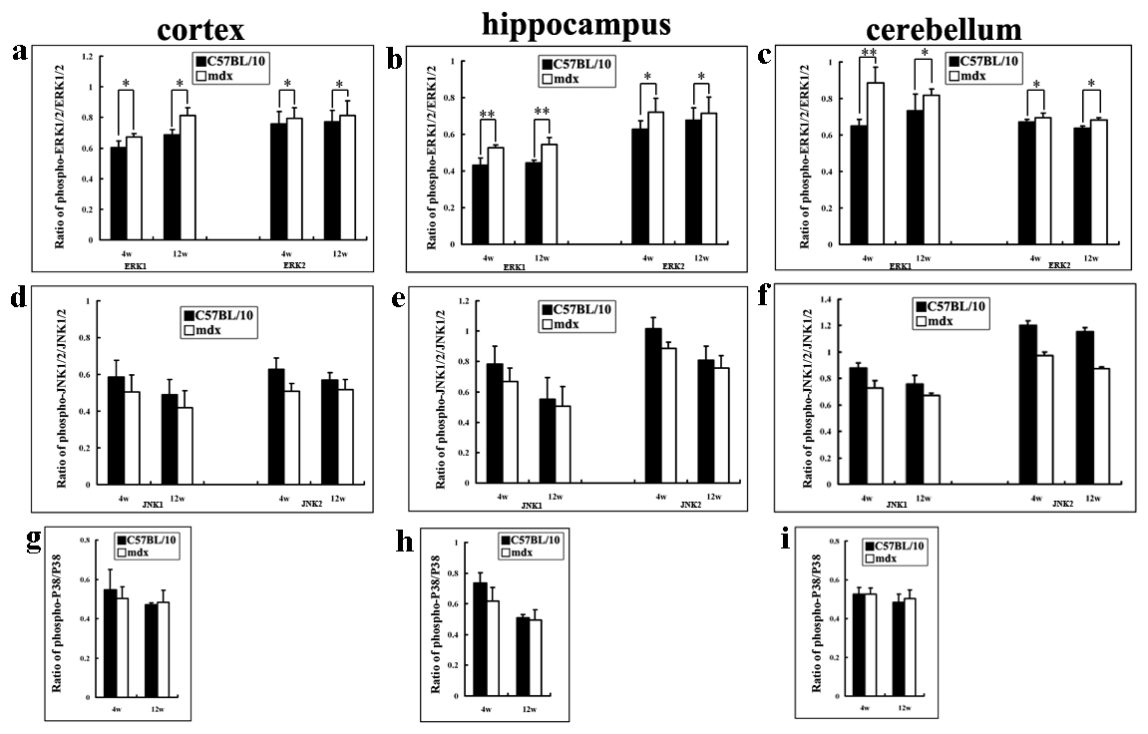
OD data for MAP kinases in the cerebral cortex, hippocampus and cerebellum in mdx and C57BL/10 mice aged 4 and 12 weeks. Data were shown as the ratio of phosphorylated and non-phosphorylated kinase levels. Levels of p-ERK1/2 in all areas were higher in mdx mice than in C57BL/10 mice at both 4 and 12 weeks (**a**–**c**). No differences in the phosphorylation levels of JNK1 or JNK2 between mdx and C57BL/10 mice were observed (**d**–**f**). The level of phosphorylated p38 MAPK (p-p38) also showed no difference between mdx and C57BL/10 mice (**g**–**i**). All values are the mean ± SD. *p < 0.05, **p < 0.01.

**Figure 9:**
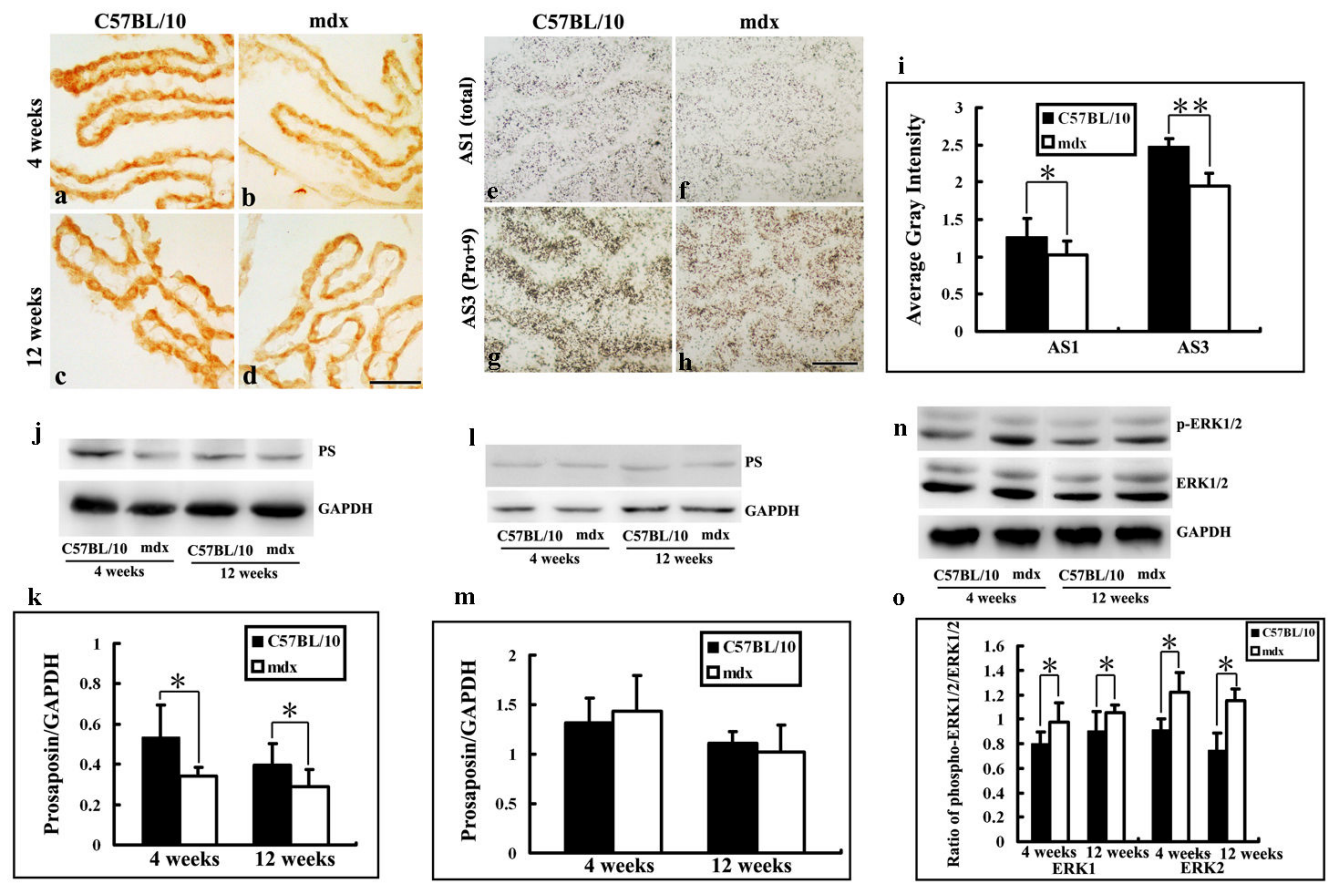
PS expression in the choroid plexus in C57BL/10 and mdx mice as detected by immunochemistry and *in* *situ* hybridization; the PS protein levels in the choroid plexus and CSF and the expression of phosphorylated and non-phosphorylated ERK in the choroid plexus of mdx and C57BL/10 mice detected by Western blot. **a**–**d**: PS immunoreactivity is present in the somas of ependymal cells in the choroid plexus in juvenile (**a**, **b**) and adult (**c**, **d**) C57BL/10 and mdx mice. Bars = 20 μm. **e**-**h**: PS mRNA expression in the choroid plexus in C57BL/10 and mdx mice at 4 weeks. Detection of total mRNA with AS1. Detection of exon 8-containing PS mRNA with AS3. AS1 and AS3 showed intense signals in C57BL/10 mice, but weak signals in mdx mice**. i**: Results were analyzed by ANOVA followed by Fisher’s post-hoc test and are presented as a histogram (*p < 0.05). Bars = 10 μm. **j**, **l**: Western blot analysis showing PS as a 65-kDa protein in the choroid plexus and CSF of juvenile and adult C57BL/10 and mdx mice. **k**: Relative PS protein levels in the choroid plexus of mdx and control mice at 4 and 12 weeks. **m**: Relative PS protein levels in the CSF of mdx and control mice at 4 and 12 weeks. Densitometric values were normalized using GAPDH as an internal control. Results were analyzed using Fisher’s post-hoc test (*p < 0.05). **n**: Western blot showing the expression of phosphorylated and non-phosphorylated ERK in the choroid plexus of mdx and C57BL/10 mice aged 4 and 12 weeks. **o**: Levels of p-ERK1/2 were higher in mdx mice than in C57BL/10 mice at both 4 and 12 weeks. All values represent means ± SD. *p < 0.05.

All of the data obtained by Western blotting are summarised as histograms in Figure 8. In the cortex, the level of p-ERK1 increased about 1.1-fold in juvenile mdx mice and about 1.2-fold in adult mdx mice compared with C57BL/10 mice of the same age. The p-ERK2 level increased about 1.05- and 1.08-fold in juvenile and adult mdx mice, respectively, compared with C57BL/10 mice of the same age (Figure 8a). In the hippocampus, the level of p-ERK1 increased about 1.22-fold in juvenile mdx mice and about 1.16-fold in adult mdx mice compared with C57BL/10 mice of the same age (Figure 8b). The p-ERK2 level increased about 1.22- and 1.06-fold in juvenile and adult mdx mice, respectively, compared with C57BL/10 mice of the same age (Figure 8b). In the cerebellum, the level of p-ERK1 increased about 1.36- and 1.1-fold in juvenile and adult mdx mice, respectively, compared with C57BL/10 mice of the same age (Figure 8c); the p-ERK2 level increased about 1.04- and 1.08-fold in juvenile and adult of mdx mice, respectively, compared with C57BL/10 mice of the same age (Figure 8c). The level of p-ERK1 and p-ERK2 was also analyzed in the choroid plexus. The p-ERK1 level increased 1.22-fold in juvenile mdx mice and 1.16-fold in adult mdx mice compared to C57BL/10 mice of the same age, while the p-ERK2 level increased 1.32- and 1.33-fold in juvenile and adult mdx mice, respectively, compared to C57BL/10 mice of the same age (Figure 9n-o).

### Secreted PS levels in mdx and C57BL/10 mice

Most CSF is produced in the brain by modified ependymal cells in the choroid plexus and the remainder is formed around blood vessels and along ventricular walls. PS exists as a secretory protein in CSF and its expression is concentrated in epithelial cells of the choroid plexus [1,8,29]. To investigate the secreted levels of PS in our study, PS expression in the choroid plexus and CSF were analyzed by immunohistochemistry, Western blotting, and *in situ* hybridization. 

In the choroid plexus of C57BL/10 mice, PS immunoreactivity was predominantly present in the somas ependymal cells in animals aged 4 and 12 weeks (Figure 9a–d). The PS staining pattern was similar but less intense in mdx mice of both ages (Figure 9b, d). This was confirmed by *in situ* hybridization (Figure 9e-h). The hybridization signals for total mRNA (AS1, Pro+9 and Pro+0) were weaker in mdx mice than in C57BL/10 mice (1.02 ± 0.09 vs. 1.27 ± 0.23; Figure 9i). The same was true for Pro+9 mRNA (AS3, encoding secretory-type PS; 1.95 ± 0.16 vs. 2.48 ± 0.19; Figure 9h). 

Densitometry of PS-immunoreactive bands showed that PS levels were significantly lower in the choroid plexus of mdx mice than control mice at 4 weeks (0.34 ± 0.04 vs. 0.53 ± 0.15, *p* < 0.05) and 12 weeks (0.29 ± 0.08 vs. 0.39 ± 0.11, *p* < 0.05; Figure 9g-k). However, there were no differences in the PS level in CSF between mdx mice and C57BL/10 mice of either age (Figure 9l-m).

### GPR37 and GPR37L1 expressions in mdx and C57BL/10 mice

**Figure 10:**
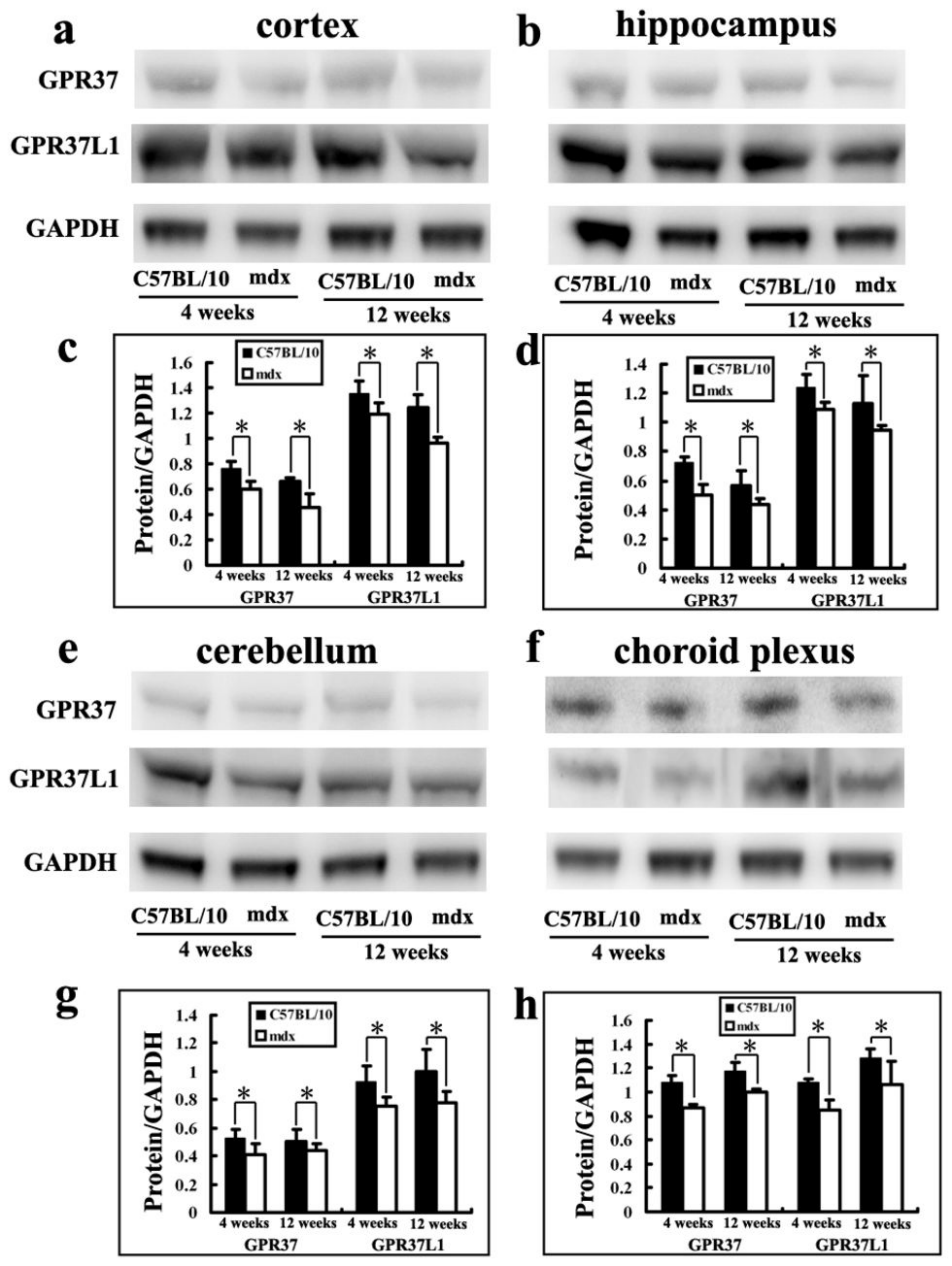
GPR37 and GPR37L1 expression in mdx and C57BL/10 mice aged 4 and 12 weeks. **a**: Cerebral cortex. **b**: Hippocampus. **e**: Cerebellum. **f**: Choroid plexus. GPR37 and GPR37L1 were analyzed by Western blotting. GAPDH was used as a control for protein loading. **c**-**d**, **e**-**f**: Optical density data for GPR37 and GPR37L1 showed that the signal for GPR37L1 is stronger than GPR37 in brain tissues. GPR37 and GPR37L1 were significantly lower in cortex, hippocampus, cerebellum and choroid plexus of mdx mice than those of control mice at 4 weeks and 12 weeks. All values represent means ± SD. *p < 0.05.

A recent report identified PS and prosaptide as ligands for the orphan receptors GPR37 and GPR37L1 [30] and showed that GPR37 and GPR37L1 mediate protective actions of secreted PS. In our study, the expression of these receptors was detected in the cortex, hippocampus, cerebellum, and choroid plexus of mdx and C57BL/10 mice by Western blot (Figure 10a-b, e-f). Densitometry of immunoreactive bands showed stronger signals for GPR37L1 than GPR37 in brain tissues (Figure 10a-b, e-f). In the cerebral cortex, GPR37 expression was significantly lower in mdx mice than in control mice at 4 weeks (0.59 ± 0.06 vs. 0.76 ± 0.05, *p* < 0.05; Figure 10c) and 12 weeks (0.45 ± 0.11 vs. 0.66 ± 0.02, *p* < 0.05; Figure 10c), as was GPR37L1 expression at 4 weeks (1.19 ± 0.09 vs. 1.35 ± 0.10, *p* < 0.05; Figure 10c) and 12 weeks (0.96 ± 0.04 vs. 1.24 ± 0.09, *p* < 0.05; Figure 10c). In the hippocampus, GPR37 expression was lower in mdx mice than in C57BL/10 mice at both 4 weeks (0.5 ± 0.07 vs. 0.72 ± 0.03, *p* < 0.05; Figure 10d) and 12 weeks (0.43 ± 0.04 vs. 0.56 ± 0.12, *p* < 0.05; Figure 10d). GPR37L1 expression was significantly lower in mdx mice than in control mice at 4 weeks (1.08 ± 0.04 vs. 1.24 ± 0.08, *p* < 0.05; Figure 10d) and 12 weeks (0.94 ± 0.03 vs. 1.12 ± 0.19, *p* < 0.05; Figure 10d). Similar results were obtained in the cerebellum. GPR37 expression was lower in mdx mice than in C57BL/10 mice at both 4 weeks (0.41± 0.08 vs. 0.53 ± 0.06, *p* < 0.05; Figure 10g) and 12 weeks (0.44 ± 0.04 vs. 0.54 ± 0.08, *p* < 0.05; Figure 10g), and GPR37L1 expression was significantly lower in mdx mice than in control mice at 4 weeks (0.75 ± 0.06 vs. 0.92 ± 0.11, *p* < 0.05; Figure 10g) and 12 weeks (0.78 ± 0.07 vs. 0.99 ± 0.15, *p* < 0.05; Figure 10g). In the choroid plexus, the expressions of GPR37 and GPR37L1 were both lower in mdx mice than in control mice at 4 weeks (GPR37: 0.86 ± 0.03 vs. 1.08 ± 0.05, *p* < 0.05; GPR37L1: 0.85 ± 0.08 vs. 1.05 ± 0.02, *p* < 0.05; Figure 10h) and 12 weeks (GPR37: 1.00 ± 0.03 vs. 1.17 ± 0.07, *p* < 0.05; GPR37L1: 1.06 ± 0.19 vs. 1.28 ± 0.06, *p* < 0.05; Figure 10h).

### Associating MAPK and PS in SH-SY5Y cells

**Figure 11:**
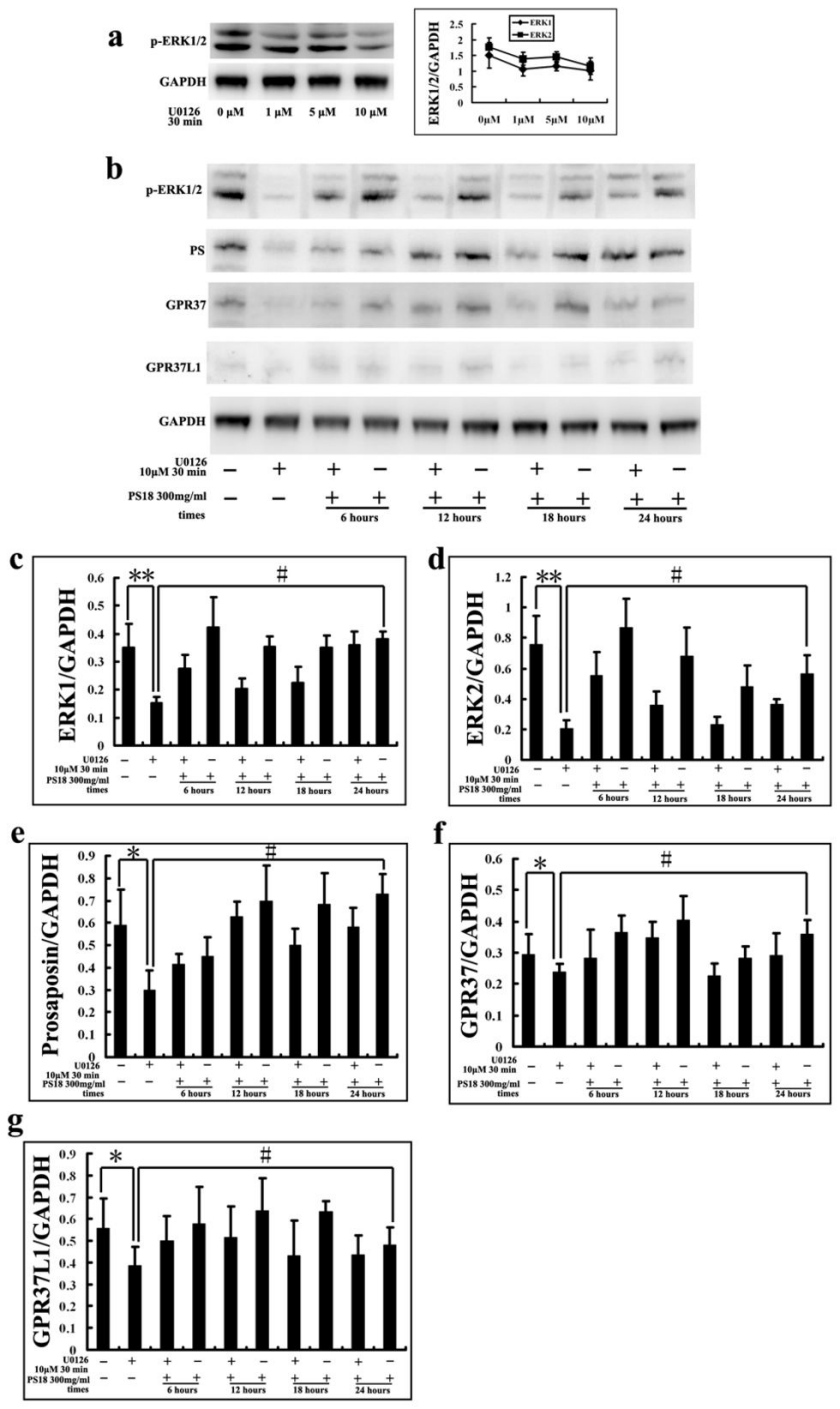
ERK, PS and PS receptors, GPR37 and GPR37L1, were analyzed by Western blotting (a-b). SH-SY5Y cells were treated with different concentrations of U0126 (1–10 μM) for 30 min to optimize the experimental conditions. U0126 (10 μM, 30 min) selectively inhibited p-ERK1/2 (**a**). SH-SY5Y cells were pretreated with 10 μM U0126 for 30 min prior to 300 ng/mL PS18 for 6–24 h (**b**). (**c**-**g**): The levels of ERK, PS, GPR37 and GPR37L1 were quantified by densitometric analysis normalized using GAPDH as an internal control. Quantitative densitometry analysis was performed using NIH Image J software. Cells were collected (three independent wells of each group) and each experiment was repeated three times. All values are means ± SD. *p < 0.05, **p < 0.01 compared to the control group (Fisher’s post hoc test); #*p* < 0.05 compared to the U0126 group.

To gain further insight into the connection between p-ERK and PS, the specific MEK1/2 inhibitors U0126 and PS18 were used in *in vitro* experiments. SH-SY5Y neuroblastoma cells were exposed to different concentrations of U0126 (1–10 μM) for 30 min to optimize the experimental conditions. U0126 at 10 μM selectively inhibited p-ERK1/2 (Figure 11a). In a previous study [4], the addition of 300 ng/mL PS18 to SH-SY5Y cells did not exhibit significant toxic effects. Thus these concentrations were used to evaluate whether ERK activation was related to PS expression. Cells were pretreated with 10 μM U0126 for 30 min prior to adding PS18. After culturing in the presence of PS18 or DMEM for 6, 12, 18, or 24 h, cells were collected and analyzed by Western blot. Interestingly, pretreatment with U0126 markedly suppressed PS expression and its receptors, GPR37 and GPR37L1 (*p* < 0.01, Figure 11b, e-g). However, after treatment with PS18, these levels increased to different degrees after 6–24 h (*p* < 0.05, Figure 11d-g). In addition, the levels of p-ERK1/2 in SH-SY5Y cells were stimulated with PS18, peaking 6 h after treatment (Figure 11c-d). These results demonstrate that ERK1/2 activity is positively correlated with PS activity, and PS18 activates p-ERK1/2 in SH-SY5Y cells.

## Discussion

The data presented here provide a comprehensive picture of the distribution of PS in the mouse brain. The spatio-temporal expression of PS differed between mdx and C57BL/10 mice, indicating that DMD is not only related to muscles but also to the CNS in mdx mice. 

 In human DMD, muscle weakness begins at age 3–4 years. This muscle weakness is due to irreversible, progressive loss of skeletal muscle and results in the need for a wheelchair at age 10 years and death at 20 years. The pathology of the mdx mouse is characterised by histologically well-defined stages with similarity to the human pathology. Necrotic or apoptotic processes in combination with inflammation emerge at approximately 3 weeks of age [23]. Regeneration processes are initiated around the age of 6 weeks and continue, together with ongoing degeneration, until 12 weeks of age [31-34]. PS levels in mdx-affected muscle decreases at these ages [7]. Thus, in our study, we used mdx mice aged 4 and 12 weeks.

 Patients with DMD display a variable degree of cognitive impairment, ranging from mild deficits in verbal skills, selective attention and and poor memory performance to mental retardation [19,35,36]. Genetic loss of dystrophin has long been suggested to be responsible for some of these deficits, as dystrophin is normally expressed in brain structures involved in diverse cognitive functions, such as the hippocampus, neocortex and cerebellum [25,37], areas where PS is abundant.

 PS potently facilitates regeneration in ischemic hippocampal neurons and transected sciatic nerves [38,39]. *In vivo* studies showed that PS and PS-derived peptides prevent ischemia-induced hippocampal neuronal death and ameliorate subsequent learning disabilities [40,41]. PS-derived peptides also prevented neuronal loss in MPTP-induced Parkinson’s disease [4,42]. In the present study, PS was expressed in neurons in mdx and C57BL/10 mice but showed reduced levels of expression in many brain regions, suggesting that PS may be related to some pathological changes in the brains of mdx mice.

Dystrophin and its autosomal homolog utrophin (Utrn) form the DAPC, which effectively forms transmembrane links between the extracellular matrix and the cytoskeleton [43,44]. The amino and carboxy termini of Utrn and dystrophin share considerable amino acid sequence homology with actin- and dystroglycan-binding domains [45,46]. DAP reduction is associated with alterations of the blood-brain barrier (BBB) [47,48] during development of the dystrophic mdx mouse. PS is present in CSF and neuronal tissues and prevents apoptosis of neuronal cells [38,40,49,50]. Western blot and immunohistochemistry demonstrated that PS levels decreased in the mdx brain. A lack of dystrophin may induce neuronal and/or BBB damage and may be related to PS expression, while the decreased level of PS may affect neuronal function.

Secretory-type PS mRNA (Figure 9e-h) in the choroid plexus and PS protein in both the choroid plexus (Figure 9a-d, j) and CSF (Figure 9l) were detected in mdx and C57BL/10 mice. In both juvenile and adult mice, mRNA and protein expression were decreased in the choroid plexus of mdx mice, but protein levels in CSF were similar. This discrepancy may be explained by the low level of PS receptors in the brain (Figure 10), whereas PS in the CSF is normal (Figure 9l-m) regardless of low PS production in the choroid plexus (Figure 9j-k).

 The highest levels of exon 8-containing PS mRNA were detected in the brain, heart and skeletal muscle [51-55]. Exon 8-containing PS mRNA is translated to unprocessed PS, which is more efficiently secreted, whereas PS mRNA without exon 8 is translated to the PS precursor of the four lysosomal saposins. Several studies have demonstrated a sharp decline in the exon 8-containing PS isoform in the rat brain following ischemia and stab wounds [53]. In our study, cRNA probes recognising total PS (AS1), secretory-type (AS3) or lysosome-type PS (AS4) allowed us to show the cellular localisation of PS mRNA isoforms in defined areas of the brain. Based on *in situ* hybridization data, we detected prominent expression of Pro+9 mRNA in juvenile and adult brain tissues, suggesting that exon 8-containing secretory-type PS is expressed in these neurons. At the same time, we found that full-length PS is also expressed in brain regions. Furthermore, we showed that PS-positive cell numbers decreased in juvenile and adult mdx mouse brains. Recent research showed that the expression level of exon 8-containing PS mRNA in mice reaches a peak after birth, when synaptogenesis is extensive [51]. Changes in synaptic transmission have been well documented to be associated with neurotoxicity after nerve injury [56,57]. In DMD, the cognitive impairment in the nervous system may be associated with the decrease in PS levels.

GPR37 and GPR37L1 are orphan G protein coupled receptors, almost exclusively expressed in the nervous system [30], for the neuroprotective and glioprotective factors prosaptide and PS. Prosaptide stimulation of cells transfected with GPR37 or GPR37L1 induces the phosphorylation of ERK. As demonstrated by our data, when the activation of ERK was inhibited by U0126, expression of PS and these receptors was inhibited (Figure 11e-g), and PS18 stimulated ERK phosphorylation (Figure 11c-d) in SH-SY5Y cells. However, interestingly, in the brains of mdx mice, the expressions of PS and its receptors (Figure 10) were decreased despite an increase in ERK (Figures 7, 8). This indicates that regulation of the ERK pathway is complex in the brains of mdx mice.

 The MAPK family is an important mediator of signal transduction processes that coordinate the cellular response to a variety of extracellular stimuli. Three major mammalian MAPK subfamilies have been described: ERK, the c-Jun N-terminal kinases (JNK, also called stress-activated protein kinase), and the p38 kinases. Each MAPK is activated through a specific phosphorylation cascade. ERK activation controls various cell responses, such as proliferation, migration, differentiation and death [58]. Many studies have supported the general view that activation of the ERK pathway delivers a survival signal [28] and our *in vitro* experiment demonstrated that PS18 activated p-ERK1/2 in SH-SY5Y cells. This is similar to what happens when PS and prosaptides (peptides encompassing the neurotrophic region of PS) bind to a putative G protein-coupled receptor [59] and activate ERK [27]. Interestingly, in our *in vivo* study, there were no changed in JNK and p38 expression but increased in ERK1/2 expression in the brains of mdx mice (Figures 7, 8, 9h). Activation of ERK1/2 has been demonstrated in mdx-affected skeletal muscle [60]. Furthermore, ERK activity can promote either intrinsic or extrinsic apoptotic pathways by inducing mitochondrial cytochrome c release or caspase-8 activation, permanent cell cycle arrest, and/or autophagic vacuolization [61]. ERK activity has been clearly implicated in neurodegenerative diseases and brain injury following ischemia/reperfusion in rodents [62-64]. The Ras/Raf/ERK pathway plays a critical role in promoting several forms of cell death in response to numerous stress stimuli *in vitro* and *in vivo*. From these reports and our studies, we speculate that activated ERK may contribute to apoptosis in the brains of mdx mice and further decrease the expression of PS and its receptors. The precise mechanisms responsible for these findings should be investigated in further studies.

 In summary, PS expression was lower in the brains of mdx mice, indicating that PS is associated with dystrophin deficiency. However, the mechanisms underlying dystrophin deficiency and these decreased PS levels remain to be determined. Further work may be focused on ERK phosphorylation and apoptosis in mdx mice, and the neuroprotective actions of PS and prosaptide mediated by GPR37 and GPR37L1 that may provide new therapeutic possibilities for the treatment of DMD.

## Materials and Methods

### Animals

Male mdx mice (C57BL/10-mdx; Clea Japan Inc., Tokyo, Japan) aged 4 or 12 weeks and C57BL/10 (Clea Japan Inc., Tokyo, Japan) of the corresponding ages were used in this study. All animals were housed at a constant temperature (22°C) under a 12/12 h light/dark cycle and given food and water ad libitum. This study was carried out in strict accordance with the recommendations in the Guidelines of the Animal Care Committee of Ehime University. The protocol was approved by the Animal Care Committee of Ehime University (Permit Number: 05A261). All surgery was performed under sodium pentobarbital anesthesia, and all efforts were made to minimize suffering.

### Prosaposin Antibody and 18-mer peptide

Medical and Biological Laboratories Co., Ltd (Nagoya, Japan) performed all of the procedures to create the PS-specific antibody (PS-Ab). Analysis of the amino acid sequence of rat PS (M19936 [65]; showed that an antibody specific for PS could be generated by immunising rabbits with a synthetic oligopeptide corresponding to 409-PKEPAPPKQPEEPKQSALRAHVPPQK-434, a portion of PS that undergoes proteolysis to generate four saposins. This amino acid sequence does not encode any saposin, and the analysis included protein secondary structure predictions and analyses of accessibility to solvents, flexibility, surface probability, antigenicity and hydrophilicity, as well as dipole analyses. The analytical method involved 150 g of conjugate in 500 L of phosphate-buffered saline (PBS), emulsified with complete Freund’s adjuvant and injected subcutaneously into a rabbit. Five booster immunisations of emulsions in incomplete Freund’s adjuvant followed at 4–8-week intervals. The rabbit was killed and bled 10 days after the final injection. The antiserum was affinity-purified with the oligopeptide. The PS-Ab titre in the serum was 1:10 000 in Western blot analyses. The species reactivity was also confirmed in mice by Western blotting. An 18-mer peptide (PS18: LSELIINNATEELLIKGL) comprising the hydrophilic sequence of rat saposin C was synthesized by Operon Technology (Tokyo, Japan).

### Cerebrospinal fluid withdrawal

Cerebrospinal fluid (CSF) samples are taken from the cisterna magna using a method that was published previously [66]. In brief, the mouse was euthanized then placed prone on the stereotaxic instrument and the head was secured with the head adaptors, the posterior neck muscles were removed with a surgical blade and a glass capillary tube with the inner diameter of about 0.5 mm (Borosilicate glass, B100-75-10, The Sutter Instrument Inc) was inserted through the arachnoid membrane into the cisterna magna. CSF was aspirated by capillary forces. Repeated specimens (obtained in three to four suctions from the same opening) were examined for the visible presence of blood by comparing small CSF sample in the pipette to a brightly lit white background. Any discrepancy between pipette colour and white surface was used as a criterion to discard the last specimen and terminate sampling. Approximate 10–20 μl of clean CSF was obtained from each mouse. CSF was collected in 500 μl tubes and subsequently stored at -80°C until use.

### Immunohistochemical staining for PS

Three mice in each group were transcardially perfused with saline, followed by 4% paraformaldehyde. Their forebrains and cerebellums were dissected and immersed in the same fixative at 4°C. The samples were then dehydrated and embedded in paraffin. Serial 7-µm coronal sections were cut using a microtome. The routine avidin-biotin complex (ABC) method was used to detect the distribution of PS in the rat hippocampus and cortex. Briefly, sections were dewaxed, rehydrated and treated with 0.1 M PBS containing 10% methanol and 3% hydrogen peroxide (H_2_O_2_) for 10 min. After rinsing with PBS, the sections were treated with 5% bovine serum albumin (BSA), 1% normal swine serum (NSS) and 1% normal goat serum (NGS) in PBS for 1 h and then incubated overnight with rabbit anti-PS (1:100) at 4°C. After rinsing, the sections were incubated in biotinylated goat anti-rabbit IgG (1:500) for 2 h at room temperature. After rinsing, the avidin–biotin–peroxidase complex (1:300; Dako, Glostrup, Denmark) was applied for 1 h at room temperature. The sections were immersed in 3,3-diaminobenzidine (Sigma, St. Louis, MO, USA) with 0.0033% H_2_O_2_ for about 10 min. After rinsing with distilled water, the sections were mounted and examined under a light microscope. As a negative control, some sections were incubated with normal rabbit serum (1:100) instead of the primary antibody and processed as described above. Nonspecific staining was not observed.

### Western blotting

Three mice were euthanised by intraperitoneal injection of an overdose of sodium pentobarbital. The cerebral cortex, hippocampus, cerebellum and choroid plexus were dissected. Briefly, the tissues were homogenised 1:5 (w/v) in ice-cold lysis buffer containing 50 mM Tris-HCl (pH 7.4), 150 mM NaCl, 1% Nonidet P-40, 1 mM ethylenediaminetetraacetic acid (EDTA), 0.25% sodium deoxycholate, 0.1% sodium dodecyl sulphate (SDS), protease inhibitor cocktail and phosphatase inhibitor cocktail (both 1:100; Nacalai Tesque, Kyoto, Japan). The resulting homogenates were centrifuged (12 000 × *g*, 30 min, 4°C). The supernatants were collected, and total protein levels were determined using a BCA protein assay kit (Pierce, Rockford, IL, USA). CSF was withdrawal by the pervious method. Proteins (15 μg) and 2μl undiluted CSF in LDS sample buffer were separated on 12% SDS polyacrylamide gels and transferred onto polyvinylidene difluoride (PVDF) membranes in a wet transfer device (30 V, 1 h). Membranes were preincubated in 5% BSA for 2 h and then incubated overnight at 4°C with the following primary antibodies: rabbit anti-PS-Ab (1:500), rabbit anti-phospho-ERK1/2 (Thr202/Tyr204; 1:500; EnoGene Biotech, New York, NY, USA), rabbit anti-ERK1/2 (1:500; EnoGene Biotech), rabbit anti-phospho-p38 (Tyr182; 1:500; EnoGene Biotech), rabbit anti-p38 (1:500; EnoGene Biotech), rabbit anti-phospho-SAPK/c-Jun N-terminal kinase (JNK; 1:1000 Thr183/Tyr185, 9251; Cell Signaling Technology, Danvers, MA, USA), rabbit anti-SAPK/JNK (1:1000, 9252; Cell Signaling Technology), Rabbit anti-GPR37 (1:500; Abnova Technology, Taipei, Taiwan) and rabbit anti-GPR37L1 (1:1000, Abnova Technology) and a mouse anti-GAPDH polyclonal antibody (1:1000; Imgenex, San Diego, CA, USA). Membranes were washed and incubated with horseradish peroxidase-conjugated secondary antibodies (1:5000; KPL, Gaitherburg, MD, USA) against rabbit or mouse for 1 h. After washing, the membranes were reacted with reagents from an enhanced chemiluminescence (ECL) kit (New England Lab, Woburn, MA, USA). Finally, specific protein bands were visualised by exposing the membranes to film (FujiFilm, Tokyo, Japan). After development, the intensities of protein bands were quantified using ImageJ software (NIH, Bethesda, MD, USA). 

### In situ hybridization

*In situ* hybridization was performed to detect PS mRNA as previously described [67-69]. Briefly, six mice aged 4 weeks in each group were killed by decapitation. Forebrains and cerebellums were immediately dissected, frozen in dry ice and stored at -80°C. Sections (20 μm thick) were cut on a cryostat, thaw-mounted onto silane-coated slides and stored at -80°C until use. 

 Three antisense 36-mer oligonucleotide probes, AS1, AS3 and AS4, and one sense probe, SS1 (used for control), were synthesised commercially (Operon Biotechnologies, Inc., Tokyo, Japan). AS1 was complementary to bases 1704–1739 in the 3'-untranslated region of the PS cDNA, permitting the detection of both Pro+9 mRNA and Pro+0 mRNA (total PS mRNA). AS3 was synthesised to detect Pro+9 mRNA (exon 8-containing PS mRNA), as the sequence of the PS cDNA determined by Collard et al. [65] does not contain the 9-base insertion after base 801 of the PS cDNA [53] and thus only detects Pro+9 mRNA. In contrast, AS4 was complementary to bases 778–813 of the PS cDNA, which excludes the 9-base insertion, and thus detects Pro+0 mRNA (exon 8-excluded PS mRNA). The sense probe SS1, complementary to AS1, was used as a control. The sequences of the four probes were as follows:

SS1: 5’-GCAGAAGTCGCCTACTTGTGGGTCTAGGGTAATGAA-3’ (negative control)
AS1: 5’-TTCATTACCCTAGACCCACAAGTAGGCGACTTCTGC-3’ (Pro+0 and Pro+9)
AS3: 5’-CTTGGGTTGCTGATCCTGCATGTGCATCATCATCTG-3’ (Pro+9)
AS4: 5’-TTCCTTGGGTTGCATGTGCATCATCATCTGGACGGC-3’ (Pro+0)

The sequence in italics (AS3) is complementary to the 9-base insertion. The underlined sequences in AS3 and AS4 are the shared sequences. The probes were labelled with ^[35S^]dATP (46.2TBq/mmol; PerkinElmer Life Sciences, Boston, MA, USA) using terminal deoxynucleotidyl transferase (Takara, Tokyo, Japan), and a specific activity of approximately 1.0 × 10^7^ dpm/ml was obtained. 

 Sections were fixed in 4% paraformaldehyde in 0.1 M sodium phosphate buffer (pH 7.4) for 15 min, rinsed in 4× standard saline citrate (SSC, pH 7.4) and dehydrated through a graded ethanol series. Sections were then hybridised with ^35^S-labelled probes in hybridization buffer (50% deionised formamide, 1% Denhardt’s solution, 250 μg/ml yeast total RNA, 0.1 g/ml dextran sulphate, 0.12 M PB and 20 mM DTT in 4× SSC) at 41°C overnight. After hybridization, sections were rinsed three times in 1× SSC at 55°C for 20 min, dehydrated through a graded ethanol series, coated with Kodak NBT-2 emulsion (Eastman Kodak, Rochester, NY, USA) and exposed at 4°C for 4 weeks. Finally, the sections were developed in a D-19 developer (Eastman Kodak). After dehydration and mounting, the sections were observed under a microscope. 

The grey intensity was examined under an Eclipse E-800M microscope (Nikon, Tokyo, Japan) coupled to a Pro-Series High Performance CCD camera (Sony, Tokyo, Japan). Scanning was performed at 400× magnification to measure the average intensity. For quantification, 10 sections per animal were analyzed and the optical density (OD) was calculated conventionally: OD = [log_10_ (incident light/transmitted light)]. The grey intensity was analyzed using ImageJ.

### Human SH-SY5Y neuroblastoma cells culture and treatment

Human SH-SY5Y neuroblastoma cells (ATCC, Manassas, VA, USA) were cultured in Dulbecco’s minimum essential medium (DMEM; Wako, Osaka, Japan) supplemented with 10% heat-inactivated fetal bovine serum (FBS; PAA Laboratories, Yeovil, Somerset, UK), 100 U/mL penicillin, and 100 U/mL streptomycin at a pH of 7.4. Culture medium was changed every 3–4 days, and cells were maintained in a humidified 5% CO2 atmosphere at 37°C and sub-cultured at a ratio of 1:20 every 7–10 days. Culture medium was changed to DMEM without FBS for 12 h before the start of each experiment. All experiments were performed using 70–80% confluent cultures. 1,4-diamino-2,3-dicyano-1,4-bis[2-aminophenylthio] butadiene (U0126, #9903, Cell Signaling Technology) was used to inhibit p44 and p42 MAP kinase activities. U0126 (5 mg) was resuspended in 1.31 mL DMSO to prepare a 10 mM stock. PS18 (25 µg/mL) was dissolved in 0.01 M phosphate-buffered saline (PBS) and filtered with a 0.22-µm-filter membrane (Millipore, Billerica, MA, USA). Different concentrations of U0126 and 300 ng/mL PS18 were diluted in SH-SY5Y medium and used immediately.

### Statistics

All values are expressed as the mean ± standard deviation (SD), and all statistical analyses were carried out using SPSS 13.0 (SPSS Inc., Chicago, IL, USA). Data were subjected to analysis of variance (ANOVA) followed by Fisher’s post hoc test. A *p*-value of <0.05 was considered significant.
